# Current status and needs of in-service training for psychiatric nurses in 24 provinces of China: a cross-sectional survey

**DOI:** 10.3389/fpsyg.2024.1376274

**Published:** 2024-07-02

**Authors:** Xiaolin Tan, Minghao Pan, Zhiying Wan, Ying Yang, Lijuan Zhang, Yu Fang, Xiaofen Li, Meiyu Shen

**Affiliations:** ^1^Department of Psychiatry, Renmin Hospital of Wuhan University, Wuhan, China; ^2^Rehabilitation Business Department of the Second Hospital of Huangshi City, Huangshi City, China; ^3^Obstetrics and Gynecology Department of Huangshi Second Hospital, Huangshi City, China

**Keywords:** in-service training, cross-sectional survey, psychiatric nurses, needs, China

## Abstract

**Aim:**

To investigate the current situation and need for post-competence training for psychiatric nurses in China and provide a reference for the development of training programs for psychiatric nurses.

**Design:**

A cross-sectional design.

**Methods:**

A cross-sectional study was conducted from August to October 2023 with 435 psychiatric nurses from 34 hospitals in 24 provinces of mainland China. A self-administered questionnaire was used for data collection. Descriptive statistics, non-parametric tests, and chi-square tests were used for data analysis.

**Results:**

The training content for psychiatric nurses is extensive, and the training load is large. Psychiatric nurses have high training demands for first aid knowledge, emergency handling ability, and anti-riot skills. Nurses with different years of experience have different training needs. The training needs of psychiatric nurses in specialized and general hospitals also different.

**Conclusion:**

The training status of psychiatric nurses is not consistent with the demand. Managers should combine this with psychiatric nurses’ own work needs to develop practical and effective training programs.

## 1 Introduction

Mental illness is a major health concern worldwide. Over the last 30 years, mental disorders have persisted in more than 14% of age-standardized years lived with disability (YLD), and have shown more than 10% of prevalence in all 21 Global Burden of Disease (GBD) regions (Li et al., 2022). YLD is described as the number of years lived in unsatisfactory health, with 1 YLD equivalent to a full year of healthy life lost due to disability or poor health (Collaborators, 2022). In China, Zhang et al. (2021) discovered that the prevalence of mental disorders in Shandong Province was 17.1% among 28,194 adults. Additionally, a national cross-sectional study that investigated the prevalence of mental disorders in 31 provinces with 32, 552 participants over the age of 18 showed that the weighted lifetime prevalence of any mental disorder (excluding dementia) was 16.6%, and the 12-month prevalence was 9.3% (Huang et al., 2019). Psychiatric nurses play an important role in providing care to diverse groups of patients with mental disorders, which range from adolescents in special care units to senior patients in hospitals and community homes; this demonstrates the complexity and diversity of nursing care in inpatient psychiatric settings (Frauenfelder et al., 2018). The burden of mental illness has a certain correlation with psychiatric nursing, and nursing plays an important role in the treatment of patients, and related studies have been reported (Hartley et al., 2020). In such a difficult working environment, it is important to improve the ability of psychiatric nurses. Learning through training is the main way to improve ability (Gehri et al., 2023).

Compared to other diseases, the treatment and nursing of patients with mental illness are more difficult. Facilitating the healing process in patients with psychiatric disorders depends on high-quality mental health care and expert psychiatric nurses. The particularity of the patients that psychiatric nurses serve determines the difference in the connotation and emphasis of their post ability from those of general nurses. Interpersonal communication skills, violence prevention skills, and legal and ethical practice skills are particularly important for the training of standardized psychiatric nurses (Moyo et al., 2022). Patients with mental illness face obstacles in “knowledge, emotion, and intention”; thus, establishing a therapeutic relationship and effective communication has become an important issue in psychiatric nursing work. Patients with mental illness often have rich symptoms, changeable emotions, and a high risk of impulsive violence, which leads to workplace violence in psychiatric departments. How to stabilize the patient’s condition to ensure the staff’s safety is knowledge that every staff member needs to master. Since the promulgation of the Mental Health Law of the People’s Republic of China, the rights and interests of patients with mental illness have received increasing attention. Therefore, it is necessary for psychiatric nurses to master relevant laws and regulations and correctly deal with ethical issues in practice.

Nurses’ standardized training refers to the professional training in clinical nursing received by nurses after they have completed college education, so that their knowledge and skills can be comprehensively improved. Although nurses’ standardized training has gradually evolved (Chen et al., 2022), that of psychiatric nurses is still in its infancy. Scholars (Moyo et al., 2022; Bush et al., 2023) have attempted to build a training program model for new psychiatric nurses. Moyo et al. (2022) proposed to develop the core competences of psychiatric nurses, such as clinical nursing ability, interpersonal communication ability, professional development ability, critical thinking, and research ability.

However, relatively few investigations and studies have been conducted on post-training evaluation, and they have not clarified the effect, method, and content of training and whether it meets nurses’ actual needs. Therefore, it is necessary to conduct a survey on the training status of psychiatric nurses to assess their satisfaction with training content, form, and duration, as well as with the job match. At the same time, collecting information about nurses’ demand and satisfaction with training may promote its effectiveness and provide a reliable basis for improving professional quality and establishing effective and feasible training programs.

## 2 Methods

### 2.1 Design

A national-wide cross-sectional study design was adopted.

### 2.2 Setting and participants

This study was conducted using purposive sampling from August to October, 2023. Nurses who had been on consecutive vacations for more than 3 months in the previous year, had severe physical or mental disorders, or were not currently working in nursing were excluded. A total of 443 questionnaires were collected. Nine questionnaires that had excessively long or short answers or that lacked authenticity were excluded, and 435 questionnaires were recovered, with an effective recovery rate of 98%.

### 2.3 Measures

This study used a self-administered electronic questionnaire. The questionnaire was discussed and formulated by 32 experts in an expert meeting based on a literature review (Chen et al., 2022; Moyo et al., 2022) and by combining it with the Chinese Nursing Association’s practice in conducting special training for psychiatric nursing. The experts were the chairmen of psychiatric nursing of the Professional Committee of the Chinese Nursing Association as well as of each province’s Nursing Association (autonomous region, municipality directly under the Central Government), all of whom had senior titles and had been engaged in psychiatric nursing-related work for more than 10 years. The questionnaire comprised two parts: basic information of the subjects, including gender, age, education, working years, hospital nature, etc. Research subjects training status and needs, the main topics such as the current training content, training duration, training frequency, training methods, their expected training content, duration, methods, etc.

#### 2.3.1 General information of psychiatric nurses

Gender, age, highest education, technical title, position, establishment, marital status, nature of the ward, type of medical institution, and years of psychiatric nursing work.

#### 2.3.2 Current status of psychiatric nurses’ on-the-job training and training needs

Whether they have participated in professional training related to psychiatric nursing organized by the Chinese Nursing Association, provincial and municipal nursing associations, or by the hospital or department where they are located; and the specific content, form, time frequency, evaluation method, and needs of training.

### 2.4 Data collection

In China, nursing quality control centers are responsible for managing all hospitals in every province. To conduct the study, at the beginning of the survey, permission and coordination was obtained from the administrator of nursing quality control centers in each province. A coordinator was arranged to be present at each nursing quality control center. This study used questionnaire survey method and Questionnaire Star platform to develop the questionnaire on the status and needs of in-service training of psychiatric nurses. The quick response code (i.e., two-dimensional bar code) of the questionnaire was sent to all coordinators of the nursing quality control center, who sent it to the nursing administrators of each hospital through WeChat, a free application launched by Tencent to provide instant messaging services for smart devices. A WeChat group (a network space) was established by WeChat users for the online exchange of information. Users in a group are collectively referred to as group members. Group members can quickly send voice or short messages, videos, pictures, and text messages across the network to share information. Finally, nursing managers at 34 hospitals sent quick response codes to psychiatric nurses at their hospitals. The questionnaire has been conducted anonymously, and each person can only submit once. Questionnaires with a response time of less than 2 min are excluded, and the questionnaire can be submitted only after all questionnaires are completed, so as to ensure the completeness and validity of questionnaire filling. After the questionnaires were collected, two researchers checked and cleaned the data and manually eliminated invalid questionnaires. We collected data from August to October 2023.

### 2.5 Statistical analysis

Data were analyzed using the IBM SPSS Statistics software (Version 26.0) and described using descriptive statistics such as mean, standard deviation and percentage. Non-parametric and chi-square tests were used to compare the differences in training needs of nurses with different characteristics as well as the differences in their training status and needs. Two researchers reviewed the data.

## 3 Results

### 3.1 Participant characteristics

A total of 435 psychiatric nurses from 34 tertiary and secondary hospitals across 24 provinces participated in the study. Their mean age was 35.14 years (*SD* = 7.78), and most nurses (74.71%) were married. Nearly 87% of participants were contract nurses. Approximately 90.57% were women, and nearly 42.07% were from general hospitals. Approximately 37.01% of nurses worked in open wards, and 62.99%worked in closed wards. Most nurses (83.91%) held a bachelor’s degree. Among the participants, approximately 51.26 had intermediate titles. The largest proportion of nurses (52.41%) had worked in psychiatry for more than 10 years. Table 1 shows the participants’ demo graphic characteristics.

**Table 1:** Participants’ demographic characteristics (*n* = 435).

Characteristics	*n(%)*
Age, years, Mean ± SD	35.14 ± 7.78
Gender	
Women	394(90.57)
Men	41(9.43)
Marital status	
Married	325(74.71)
Single	102(23.45)
Divorced	8(1.84)
Widowed	0
Form of employment	
Formal	54(12.41)
Contract	381(87.59)
Ward type	
Open ward	161(37.01)
Closed ward	274(62.99)
Highest educational level	
Vocational school	10(2.3)
Junior college	48(11.03)
Undergraduate	365(83.91)
Master’s or above	12(2.76)
Professional title	
Junior nurse	60(13.79)
Senior nurse	127(29.2)
Supervisor nurse	223(51.26)
Associate chief nurse	22(5.06)
Chief nurse	3(0.69)
Nature of hospital	
General hospital	183(42.07)
Specialized hospital	252(57.93)
Years as a nurse	
<1 year	14(3.22)
1–3 years	25(5.75)
3–10 years	131(30.11)
≥10 years	265(60.92)
Years as a nurse in psychiatry	
<1 year	22(5.06)
1–3 years	28(6.44)
3–10 years	157(36.09)
≥10 years	228(52.41)

Data are *n*(%), unless otherwise indicated.

### 3.2 Training status

According to the survey, the content of training for psychiatric nurses was extensive. The top three most frequently taught topics were mental illness nursing routine (96.55%), nurse–patient communication (91.72%), and protective restraint knowledge (92.64%), and the top three least frequently taught topics were career development (52.87%), psychiatric new technology and new business (61.38%), and nursing research (41.15%). Training frequency was usually once a month, and each session lasted approximately 45–90 min. The training methods were predominantly integrated through a combination of online and offline approaches, among which on-site scenario simulations and department rotation practices were the least applied. Most post-training evaluation methods were theoretical and operational assessments, and the use of scale tools for evaluation was the least common.

### 3.3 Comparison of training status and training needs

Significant differences in the training needs of psychiatric nurses were observed. The highest frequency of training was once a month (63.22%), whereas nurses expected it to be once a quarter (49.66%). The training methods were still a combination of online and offline methods; however, the expected proportion of online training increased. Lecturers were more specialist nurses (75.4%) than head nurses (67.36%). Theoretical examination was the main evaluation method (92.87%), while most nurses expected it to be satisfaction evaluation (63.45%). With regard to the training contents, the proportion of psychiatric nursing routine, hospital illness prevention and control knowledge, and protective restraint training decreased, while that of violence prevention skills training, nursing scientific research, new technology, and business and career development training increased when training needs were mentioned; the difference was statistically significant (*p* < 0.05). No statistical difference was observed with regard to other content (*p* ≥ 0.05). Additional details are provided in Table 2.

**Table 2:** Comparison of training status and training needs.

Variable	Categories	Training status *n*(%)	Training needs *n*(%)	*X^2^*	*P*
Course length	≤45 min	48(11.03)	204(46.9)	172.845	0.000
	45–90 min	260(59.77)	207(47.59)		
	>90 min	127(29.2)	24(5.52)		
Training methods	Online	20(4.6)	95(21.84)	56.439	0.000
	Offline	34(7.82)^a^	26(5.98)^a^		
	Both	381(87.59)	314(72.18)		
Training frequency	None	2(0.46)^a^	3(0.69)^a^	129.799	0.000
	Once a year	33(7.59)^a^	47(10.8)^a^		
	Quarterly	77(17.7)	216(49.66)		
	Once a month	275(63.22)	159(36.55)		
	Once a week or more	48(11.03)	10(2.3)		
Lecturer qualification	Professor	195(44.83)^a^	200(45.98)^a^	11.215	0.024
	Clinical nurse specialism	271(62.30)	328(75.4)		
	Nursing supervisor	293(67.36)	248(57.01)		
	Doctor	246(56.55)^a^	257(59.08)^a^		
	Else	3(0.69)^a^	8(1.84)^a^		
Effect evaluation methods	Theory test	404(92.87)	272(62.53)	87.868	0.000
	Skill assessment	380(87.36)	259(59.54)		
	Satisfaction survey	311(71.49)^a^	276(63.45)^a^		
	Specific scale measurement	111(25.52)	199(45.75)		
	Objective structured clinical examination	92(21.15)	151(34.71)		
Course content	Common mental illness care	420(96.55)	313(71.95)	75.440	0.000
	Nurse patient communication skills	399(91.72)^a^	342(78.62)^a^		
	Knowledge of hospital infection prevention and control	388(89.2)	276(63.45)		
	Occupational exposure protection	382(87.82)^a^	329(75.63)^a^		
	First aid training	378(86.9)^a^	334(76.78)^a^		
	Emergency handling ability	383(88.05)^a^	351(80.69)^a^		
	Common risk factors in ward	384(88.28)^a^	314(72.18)^a^		
	Departmental risk assessment	388(89.2)^a^	301(69.2)^a^		
	Riot control skills training	341(78.39)	342(78.62)		
	Knowledge of protective constraints	403(92.64)	269(61.84)		
	Drugs commonly used in psychiatry	389(89.43)^a^	292(67.13)^a^		
	Mental Health Law	361(82.99)^a^	292(67.13)^a^		
	Nursing scientific research	230(52.87)	269(61.84)		
	New technology, new knowledge	267(61.38)	305(70.11)		
	Career development	179(41.15)	240(55.17)		
	Else	3(0.69)^a^	1(0.23)^a^		

(Annotation)^a^ means (*p* ≥ 0.05).

#### 3.3.1 Comparison of training needs of nurses with different years of experience working in the psychiatric department

The results indicated that psychiatric nurses with different years of work experience showed statistically significant differences in scores for first aid training demand and riot skills training (*p* < 0.05). Psychiatric nurses with 3–10 years of experience had the highest demand for first aid training. In terms of the demand for training in violence prevention skills, psychiatric nurses with less than 1 year of service scored the highest. Additional details are provided in Table 3.

**Table 3:** Comparison of training needs of nurses with different years of experience working in the psychiatric department.

		Training needs score (RSR)	
Variable		First aid training	Riot control skills training
Years as a nurse in psychiatry	<1 year	212.84	236.95
	1–3 years	176.00	179.82
	3–10 years	237.72	234.99
	≥10 years	210.08	209.16
*z*		10.275	9.373
*p*		0.016	0.025

#### 3.3.2 Comparison of training needs of psychiatric nurses in different hospitals

The training needs of psychiatric nurses in different hospitals also showed statistically significant differences (*p* < 0.05). Compared with psychiatric nurses in specialized hospitals, psychiatric nurses in general hospitals scored higher in first aid knowledge, accident handling, risk factor, risk assessment, violence prevention skills training, career development, new business and new technology, and scientific research training. Additional details are provided in Table 4.

**Table 4:** Comparison of training needs of psychiatric nurses in different hospitals.

	Nature of hospital (RSR)			
Training item	General hospital	Specialized hospital	*z*	*p*
Common mental illness care	221.62	215.37	−0.543	0.587
Nurse–patient communication skills	219.59	216.85	−0.245	0.807
Knowledge of hospital infection prevention and control	218.43	217.69	−0.064	0.949
Occupational exposure protection	228.57	210.32	−1.617	0.106
First aid training	231.59	208.13	−2.187	0.029
Emergency handling ability	234.23	206.21	−2.653	0.008
Common risk factors in ward	236.25	204.74	−2.894	0.004
Departmental risk assessment	231.73	208.03	−2.149	0.032
Riot control skills training	235.91	204.27	−3.077	0.002
Knowledge of protective constraints	220.09	216.48	−0.319	0.750
Drugs commonly used in psychiatry	226.53	211.80	−1.322	0.186
Mental Health Law	229.88	209.37	−1.828	0.068
Nursing scientific research	231.58	208.13	−2.083	0.037
New technology, new knowledge	238.54	203.08	−3.235	0.001
Career development	233.65	206.63	−2.401	0.016

## 4 Discussion

This study found that psychiatric nurse training covered various subjects including basic knowledge, interpersonal communication, prevention and control of hospital illnesses, emergency response ability, career development, and nursing research. At the same time, nurses were provided with training in protective restraint operation, patient accident risk assessment, staff anti-riot skills, psychiatric drug knowledge, and mental health law, which met the basic requirements of psychiatric work. Despite receiving relevant training, nurses still had a high demand for training in first aid knowledge, emergency handling, and staff riot control. The scores for first aid training and riot-knowledge requirements of psychiatric nurses with different years of experience differed. The training needs scores of psychiatric nurses with 3–10 years and less than 1 year of service were higher than those of nurses with 1–3 years and more than 10 years of service. At the same time, the training requirements of psychiatric nurses in general hospitals are generally higher than those of nurses in specialized psychiatric hospitals, especially for first-aid ability, emergency handling ability, anti-riot skills training, risk factor assessment, career development, and scientific research training.

### 4.1 The expectation of post-training for psychiatric nurses is low, and their willingness to train is weakened

The results of this study showed that an increasing number of psychiatric nurses wanted less frequent training, a shorter training time, and less offline training time. The reasons may be as follows. Due to the complexity of the work environment, psychiatric nurses face substantial pressure, and they hope to reduce the burden and stress from job training. According to a survey, the turnover intention of Chinese psychiatric nurses is as high as 20.2%, and it is related to the frequent workplace violence, high work pressure, long working hours, and low respect from the patients. In addition to normal working hours, psychiatric nurses also need to undergo training, continuing education, and examinations, which often occupy their rest time and further increase their workload (Jiang et al., 2019); for nurses, this results in lower satisfaction with training. Similarly, research in Brazil has shown that mental health workers are overloaded (Hilgert et al., 2018). Therefore, the following problems should be considered in the improvement of training plans: a complete training infrastructure, sufficient time for training, and substantial practical guidance (Mitchell et al., 2022). From the perspective of training methods, the proportion of online training has increased significantly. Perhaps because the online meeting schedule is more flexible, the time for offline centralized teaching is reduced, and nurses can follow the course comfortably at home. Therefore, to achieve the training effect and improve nurses’ enthusiasm and initiative, managers should proceed by considering their actual circumstances, comprehensively considering their will and conducting targeted training rather than blindly pursuing a wide and complete approach, to effectively reduce their training load.

### 4.2 Psychiatric nurses have a high demand for training in first aid knowledge, emergency management, and prevention of violent injuries

Nurses’ ability to recognize the symptoms of acute psychosis and properly assess urgency during triage is one of the most important components of emergency mental health care. After receiving patients, psychiatric nurses often need to quickly assess various risks and determine the degree of urgency of the condition; thus, the observation and emergency response ability of psychiatric nurses are critical (Rajab Dizavandi et al., 2023). In this study, nurses had the highest demand for training in the emergency handling of psychiatric accidents, followed by staff training in riot control skills. The possible reasons for this are as follows: patients with mental illness often show changes in thinking, will, and emotions; their vital signs are stable, and the incidence of life-threatening incidents is lower than that in other departments. Therefore, psychiatric nurses are relatively inexperienced in emergency treatment work; it is easy for them to feel helpless when they encounter emergency events such as rescue as they cannot actively and effectively cooperate with doctors to perform emergency rescue work. Research (Sharma et al., 2022) has also mentioned that psychiatric nurses’ and doctors’ first aid training is insufficient and needs to be strengthened. Nurses felt that their first aid knowledge and abilities were inadequate, and their self-confidence was low. In the process of first aid, the nurse is an important person who cooperates with the doctor, and together, they need to be calm, decisive, agile, clear, and skilled in using various nursing treatment techniques to ensure the success of rescue. At the same time, patients with mental illness often experience accidental emergencies such as impulsive destruction, self-injury, suicide, and escape. Nurses must manage emergencies quickly and effectively; therefore, the ability to deal with accidents is necessary for psychiatric nurses. Although the training content covered emergency handling ability, the researchers felt that it was insufficient. It is thus suggested that more effective measures be taken to improve nurses’ emergency response ability.

Workplace violence (WPV) is the threat to, abuse of, or assault on health care workers in the workplace that is either explicitly or implicitly detrimental to their well-being, safety, and health. Due to psychiatric nurses’ special working environment and nursing patients, the risk and frequency of WPV are higher (Jang et al., 2022). Other research has shown that the incidence rate in some hospitals is as high as 92.7% (Schlup et al., 2021). Therefore, training in violence prevention skills for psychiatric nurses aims to ensure not only the smooth progress of work but also their own safety. It is important for psychiatric staff to adopt practical and effective training in violence prevention skills. However, no mature and effective intervention strategies to prevent workplace violence in psychiatry currently exist, and the literature is mostly based on investigative studies (Pelto-Piri et al., 2020; Pelto-Piri and Kjellin, 2021; Schlup et al., 2021). Pelto-Piri et al. (2020) and Pelto-Piri and Kjellin (2021) conducted a qualitative interview study of psychiatric service users, staff, and ward managers on social inclusion and violence prevention in inpatient care. A technical analysis of violence and aggression incidents in psychiatric inpatient care in Sweden was also conducted to understand the causes of violence and attitude of the staff, among other aspects. Qualitative studies (Niu et al., 2019) and investigative studies (Niu et al., 2019; Alyousef and Alhamidi, 2022; Alamri et al., 2023; Bekelepi and Martin, 2023) on psychiatric nurses’ attitudes toward and experiences of workplace violence have also been conducted in China, providing a reference for preventing workplace violence. However, few studies have been conducted on violence prevention intervention strategies for psychiatric nurses. This suggests that violence prevention interventions for psychiatric nurses are not yet mature, and more effective interventions need to be developed to reduce workplace violence toward psychiatric nurses.

### 4.3 Psychiatric nurses with different years of experience have different needs for first aid training and violence prevention training

The results showed that the scores of first-aid ability training needs and riot-knowledge training needs of psychiatric nurses who worked for 3–10 years and less than 1 year were higher than those of psychiatric nurses who worked for 1–3 years and more than 10 years. This may be because psychiatric nurses who have worked for less than 1 year have just entered the field and are still undergoing various kinds of training. At the same time, they are not sufficiently adapted to work and have little clinical experience and no knowledge in emergency management or violence prevention; therefore, they hope to receive more training to adapt to the changing clinical environment. The National Ministry of Health’s “Trial Measures for Standardized Training of Clinical Nurses” stipulates that undergraduate training is 1 year, and specialist training is 2 years. According to the actual situation in domestic hospitals, the standardized training time is generally 1–3 years (Ye et al., 2018). Psychiatric nurses who have worked for 1–3 years have completed theoretical training and clinical practice, accumulated certain theoretical knowledge and practical experience, and become familiar with the work flow, and they have acquired basic cognition of the handling of various emergencies; thus, their level of training demand is low. Nurses who have worked for 3–10 years further increased their clinical experience based on the above, but their continuing education training is relatively reduced. With the increase in years of experience, nurses face an increasing number of emergencies, and the training previously received is no longer enough to cope with all the difficulties. Therefore, they hope to accept new theoretical and practical first aid training and violence injury prevention training, and their training demand increases. Psychiatric nurses who have worked for more than 10 years as senior nurses and have substantial clinical experience have far more theoretical knowledge and practical experience than young nurses, and their training needs are reduced. Therefore, training strategies should be formulated to meet the different work needs of nurses with different working experience.

### 4.4 The training demand of psychiatric nurses in general hospitals is higher than that of psychiatric nurses in specialized hospitals

The results of this study showed that compared with specialized hospitals, psychiatric nurses in general hospitals had higher demand scores in first aid knowledge, accident handling, risk factor score, risk assessment score, violence prevention training, career development, new business and new technology, scientific research training, and other aspects. This difference was statistically significant, while no such difference was found in other psychiatric nursing routine demand scores. The reasons for this may be as follows. To meet the post requirements of all departments, the training content of general hospitals is universal, whereas psychiatric hospitals pay more attention to the related knowledge, skills, and qualities of psychiatric nursing, which reflects the special characteristics of psychiatry (Ye et al., 2020). At the same time, a study (Wang et al., 2022) showed that compared with the psychiatric department of general hospitals, psychiatric hospitals treated patients with complex and critical conditions in a wide range of disease categories. Moreover, as nurses accumulated substantial clinical experience more rapidly, they had relatively fewer needs for first aid knowledge, accident handling, risk factor identification, risk assessment, and violence prevention training. At the same time, compared to nurses in psychiatric hospitals, psychiatric nurses in general hospitals have more opportunities to receive knowledge in fields other than psychiatry, become more versatile talents, and have better career development prospects, whereas nurses in psychiatric hospitals are professional talents with limited career development. Therefore, psychiatric nurses in general hospitals have a higher demand for career development, new technology, and scientific research training. It is thus necessary to consider the nature of psychiatric nurses’ hospitals and their expectations to achieve better training results.

Overall, our study is meaningful. Through in-depth needs assessments, policymakers and care administrators can accurately understand what psychiatric nurses really think and feel about training. This includes not only their positive attitude toward training but also their resistance and opinions. This comprehensive understanding is essential for developing suitable training programs, as it helps us accurately capture the needs of nurses in the training content. Further, we can identify training points that may conflict with nurses’ actual job needs. When designing a training program, we can arrange the course content more specifically to ensure that the training is practical and specific, which will significantly increase nurses’ acceptance. When the training content is closely related to their daily work, nurses tend to be more motivated and engaged in learning, which further improves effectiveness of the training. In addition, a demand survey can effectively promote nurses’ in-depth understanding of the training content. By translating their knowledge into practical skills in a timely manner, nurses can apply the results of their training more effectively to their work, significantly improving quality and the level of service. Such training meets the needs of nurses in terms of individual professional development and assures continuous improvement for the entire nursing team. Furthermore, it promotes improvement in medical and health service levels. Policymakers and nursing managers must start with the actual needs of nurses, introduce practical policies to protect their interests, arrange work reasonably, avoid occupying nurses’ private time, and enable nurses to have sufficient rest time while attending training. In-service continuing education should complement college education to achieve optimal training results. Through the needs survey, we were able to understand the knowledge and skills that nurses have mastered; thus, when developing new training content, we can avoid repeating what has been learned and add new knowledge and practices in a targeted way. This approach is helpful for improving the training effect of nurses so that they can better apply their theoretical knowledge in actual practice. The purpose of continuing education is to expand knowledge, develop new practices, and encourage nurses to complete an effective transformation from theory to practice. Through the in-depth investigation of needs, we can accurately grasp the urgent needs of psychiatric nurses and implement the principle of “on-demand training, what is missing.” We believe that only this kind of targeted training can truly improve nurses’ professional skills. Skilled professional nurses will become important assistants to doctors. They can not only provide excellent professional services to patients but also ensure their safety, shorten the length of the hospital stay, and help patients recover quickly, thereby reducing their financial burden.

## 5 Conclusion

This study is the first to report on the status and demands of psychiatric nurses’ training in China. The results showed that the content of training for psychiatric nurses is extensive and has unique characteristics related to psychiatry. However, the training load is substantial, and more nurses hope to reduce it and receive targeted training according to their actual needs. The nature of the hospital in which they work and their years of experience affect nurses’ training needs. Managers should comprehensively consider various factors when designing training programs. First of all, it is necessary to conduct a demand survey before formulating a training program to understand what psychiatric nurses really want to be trained and learn, and to focus on the key points rather than systematizing all the contents. Secondly, training duration, mode and frequency should be designed according to nurses’ training wishes. Ensure learning effectiveness while minimizing the occupation of rest time. Thirdly, the training needs of nurses with different qualifications and working years and psychiatric nurses in different hospitals are different, and programs need to be formulated according to specific needs. Finally, the effect evaluation after training is very important, and nursing managers should adopt a variety of evaluation methods to evaluate the learning effect to ensure that the training is valuable and not a waste of time. It is hoped that more empirical studies on the training of clinical nurses in psychiatric departments will be conducted in the future.

## 6 Limitations

There are some limitations to this study. First, the data only reflect the results over the time period in which we conducted the study. Second, our sample included only psychiatric nurses in tertiary or secondary public hospitals, excluding psychiatric nurses in private hospitals. Further studies should recruit psychiatric nurses from all hospitals and collect the most recent data for analysis. In this study, we used a self-administered questionnaire as the data collection tool. Although this method was convenient and practical, we acknowledge the potential for some bias. With such a tool, the respondents’ subjectivity affects the accuracy of the data. Our questionnaire did not fully cover all aspects of self-management, possibly restricting the promotion and application of the research results. Due to the limitations of time and human resources, only quantitative data collection methods were used in this study, which affected the universality of the research results to a certain extent. In future studies, we plan to use a combination of qualitative and quantitative research methods to collect data and improve the universality and accuracy of our results.

## 7 Relevance to clinical practice

This study found that the training needs of psychiatric nurses are many; however, to reduce the training load, managers need to formulate training programs that meet the needs of clinical nurses from their real-life circumstances.

## Data availability statement

The raw data supporting the conclusions of this article will be made available by the authors, without undue reservation.

## Ethics statement

This study conforms to the principles outlined in the Declaration of Helsinki and has been approved by the Ethics Committee of Renmin Hospital of Wuhan University (Study ID: WDRY2023-K126). Informed consent was obtained from all the participants. The studies were conducted in accordance with the local legislation and institutional requirements. The participants provided their written informed consent to participate in this study.

## Author contributions

XT: Conceptualization, Data curation, Investigation, Methodology, Writing – original draft. MP: Supervision, Writing – original draft. ZW: Methodology, Project administration, Writing – review & editing. YY: Data curation, Formal analysis, Methodology, Project administration, Writing – review & editing. LZ: Data curation, Formal analysis, Methodology, Project administration, Writing – review & editing. YF: Investigation, Methodology, Project administration, Supervision, Writing – review & editing. XL: Data curation, Investigation, Project administration, Supervision, Writing – original draft, Writing – review & editing. MS: Data curation, Funding acquisition, Investigation, Methodology, Project administration, Resources, Supervision, Writing – original draft, Writing – review & editing.
